# Immunophenotype in acute exacerbation of chronic obstructive pulmonary disease: a cross-sectional study

**DOI:** 10.1186/s12931-022-02058-x

**Published:** 2022-05-28

**Authors:** Xiao-feng Xiong, Min Zhu, Hong-xia Wu, Li-li Fan, De-yun Cheng

**Affiliations:** 1 Department of Respiratory and Critical Care Medicine, West China Hospital, Sichuan University, NO. 37 Guoxue Alley, Chengdu, Sichuan 610041 People’s Republic of China; 2 Laboratory of Pulmonary Immunology and Inflammation, Frontiers Science Center for Disease-Related Molecular Network, Sichuan University, Chengdu, People’s Republic of China

**Keywords:** Chronic obstructive pulmonary disease, Acute exacerbation, CD4 + T cells, Immunity

## Abstract

**Background:**

Chronic obstructive pulmonary disease (COPD) is a heterogeneous disease, and the immune inflammatory response is thought to play an important role in pathogenesis. However, the immunophenotype of patients with COPD is unknown. Herein, we evaluated the immunophenotype of patients with acute exacerbation of COPD (AECOPD).

**Methods:**

A cross-sectional study was conducted in West China Hospital from September 2018 to October 2019. The proportion of CD4 + T lymphocyte subtypes (Th1, Th2, Th17 and Treg) and levels of serum cytokines in the peripheral blood of patients with AECOPD, stable COPD (SCOPD), healthy smokers (HSs)and healthy controls (HCs) were evaluated.

**Results:**

A total of 15 HCs, 19 HSs, 42 patients with SCOPD, and 55 patients with AECOPD were included. Compared to patients with SCOPD, Th1 cells, Th17 cells, Treg cell ratio, Th1/Th2 cell ratio, and the levels of C-reactive protein, interleukin (IL)-6, and IL-10 were significantly increased in patients with AECOPD (P < 0.001), while the proportion of Th2 cells was significantly reduced (P < 0.01). The proportion of Th17 cells was positively correlated with COPD Assessment Test score (r = 0.266, P = 0.009), modified Medical Research Council dyspnea score (r = 0.858, P < 0.0001), and Th1 cell ratio (r = 0.403, P < 0.0001) and negatively correlated with forced vital capacity (r = − 0.367, P = 0.009) and proportion of Th2 cells (r = − 0.655, P < 0.0001).

**Conclusions:**

The immunophenotype of patients with AECOPD shows abnormal activation of Th1, Th17, and Treg cells. There is a correlation between the proportion of Th17 cells and the severity of COPD; therefore, this may represent a novel index for the evaluation of COPD severity.

*Trial registration:* China Clinical Trials Registry, ChiCTR1800018452, registered 19 September 2018, https://www.chictr.org.cn/index.aspx.

**Supplementary Information:**

The online version contains supplementary material available at 10.1186/s12931-022-02058-x.

## Background

Chronic obstructive pulmonary disease (COPD) is a heterogeneous disease [1], which is reflected in the variable aetiology, clinical manifestations, imaging results, treatment responses and prognoses of affected patients. COPD can be divided into different phenotypes, which is helpful to guide individualized treatment and predict the prognosis [2]. Acute exacerbation of COPD (AECOPD) has a variety of phenotypes, from the initial clinical phenotype to the internal biological phenotype, and these have different pathophysiological characteristics. However, at present, there is no universal consensus on the phenotype of AECOPD, and there is no standard definition or diagnostic criteria [3, 4].

Further, the immunophenotype of patients with AECOPD has also not been clarified, and this is an important goal of clinical studies. Previous studies on T lymphocytes have mainly focused on T helper 1 cells (Th1) and T helper 2 cells (Th2) of CD4 + T cell subsets. Regulatory T cells (Treg) have also gained attention, but their expression in patients with COPD has not been fully elucidated. Most studies have shown that compared with healthy controls, the proportion of Treg cells is decreased in patients with AECOPD [5, 6] and stable COPD (SCOPD) [7–10]; however, some studies have reported conflicting results [11, 12]. Therefore, the distribution of Treg cells in COPD (especially AECOPD) remains controversial.

In recent years, researchers have paid increasing attention to another subgroup of CD4 + T cells, the T helper 17 cells (Th17), which can secrete cytokines such as interleukin (IL)-17A, IL-17F, IL-21, IL-22 and IL-23, which recruit neutrophils and maintain inflammation [13]. The expression of IL-17A is increased in the peripheral blood, lung, and bronchial mucosa of COPD patients [14, 15]. Vargas et al. [16] compared Th17 cells in the peripheral blood of COPD patients and found that Th17 cells in smokers were significantly higher than those in non-smokers. The above studies suggest that Th17 cells are closely related to COPD, but there are few studies on Th17 cells in AECOPD. In addition, few studies have evaluated the expression of all cytokine-producing T cell subsets in patients with AECOPD. Thus, the imbalance of CD4 + T cell subsets and their cytokines in patients with COPD needs to be further evaluated clinically.

We hypothesized that there is an immunophenotype with abnormal activation of CD4 + T cell subtypes in patients with AECOPD, which is correlated with clinical phenotype. In this study, the proportion of CD4 + T lymphocyte subtypes (Th1, Th2, Th17, Treg) was detected by flow cytometry, and the levels of serum cytokines were detected by liquid phase microarray to explore the immunophenotype of patients with AECOPD.

## Materials and methods

### Participants

All patients were diagnosed on the basis of clinical history, physical examination, chest radiograph, chest computed tomography, and pulmonary function tests in accordance with the clinical criteria for the diagnosis COPD by the GOLD guidelines (2017) [17]. We defined AECOPD as an acute worsening of respiratory symptoms that results in additional therapy; SCOPD as respiratory symptoms that were stable or mild, and the condition was stable for 3 months; healthy smokers (HSs) as a smoking history of ≥ 20 pack-years and normal lung function; and healthy controls (HCs) as nonsmokers with normal pulmonary function. In order to avoid bias caused by the levels of smoking exposure, we limited COPD patients with a smoking history of ≥ 20 pack-years. The key inclusion and exclusion criteria of participants are summarized in Table 1. An ever-smoker was defined as someone who had stopped smoking for at least 1 year. The study was conducted in accordance with the Declaration of Helsinki, and all participants gave written informed consent.

**Table 1:** Inclusion and exclusion criteria for the study

Inclusion criteria	Exclusion criteria
• Age ≥ 40 years	1) Had a history of systemic corticosteroid therapy (prednisone > 0.5 mg/kg or equivalent doses) within 1 month 2) Had a history of diseases that affect immune cells, such as connective tissue diseases, immunological diseases, haematological diseases, liver and renal failure 3) Had a history of asthma, allergic disease, or other clinically significant lung diseases 4) Had a history of heart failure, arrhythmia, mental disorders, or any malignancy
• SCOPD is defined as respiratory symptoms were stable or mild, and the condition was stable for 3 months	
• AECOPD is defined as an acute worsening of respiratory symptoms that results in additional therapy • HS is defined as a smoking history of ≥ 20 pack-years and normal lung function	
• HC is defined as a nonsmoker with normal pulmonary function	

*AECOPD* acute exacerbation of chronic obstructive pulmonary disease, *SCOPD* stable chronic obstructive pulmonary disease, *HS* healthy smoker, *HC* healthy control

### Study design

**Fig. 1:**
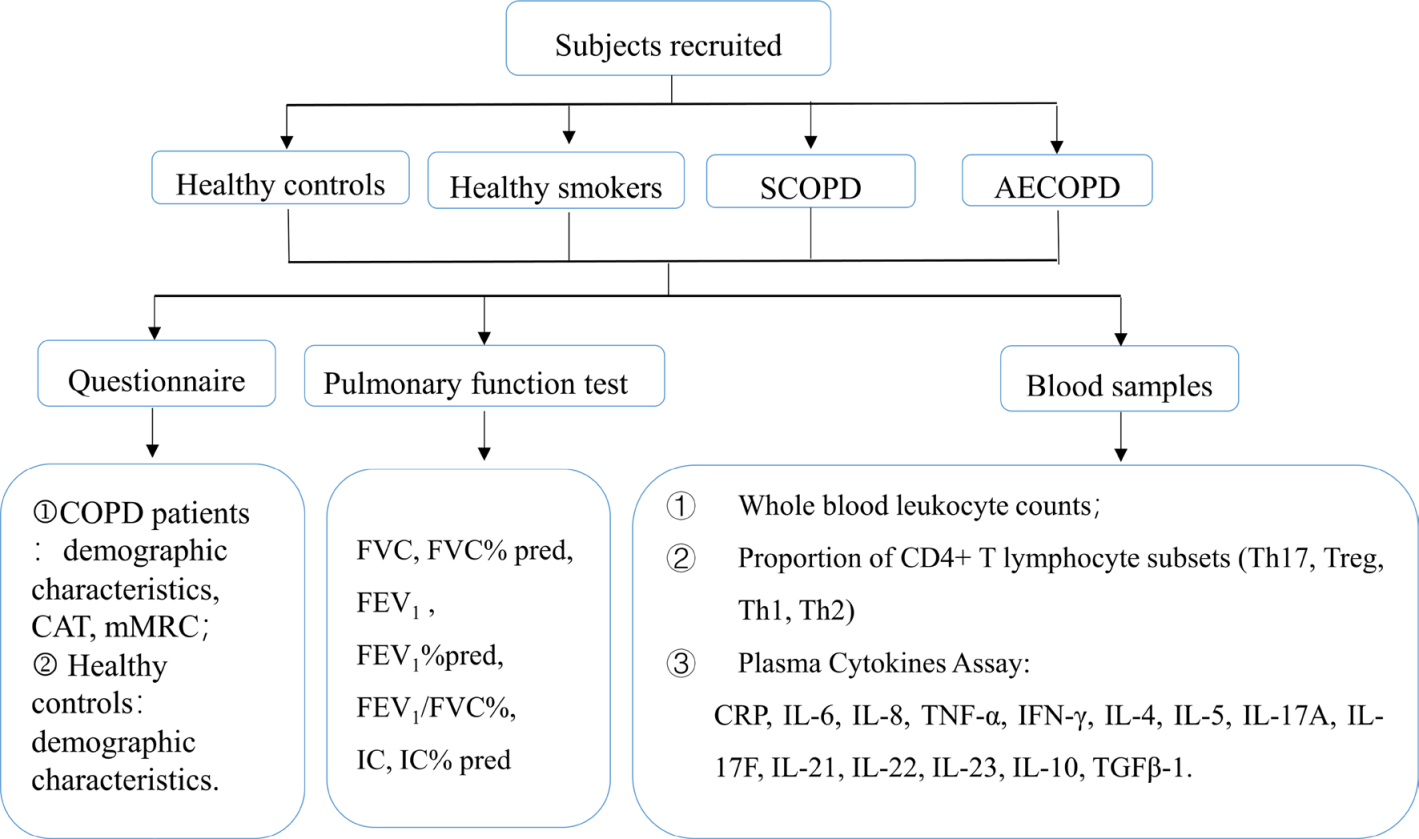
Flow diagram of the study. *AECOPD* acute exacerbation of chronic obstructive pulmonary disease, *SCOPD* stable chronic obstructive pulmonary disease, *mMRC* modified medical research council dyspnea scale, *CAT* COPD assessment test, *FEV*_*1*_ forced expiratory volume in one second, *FVC* forced vital capacity, *IC* Inspiratory capacity, *IC% pred* Inspiratory capacity % predicted

We conducted the study using a cross-sectional design. A flow diagram of the study is presented in Fig. 1. The demographic characteristics, including sex, age, body mass index (BMI), and smoking history were recorded for each participant, and pulmonary function test was also performed. Symptoms of COPD patients were evaluated according to COPD assessment test (CAT) and the modified Medical Research Council (mMRC) dyspnea scale[18].

### Flow cytometry

Flow cytometry was used to detect the proportion of CD4 + T cell subsets (Th1, Th2, Th17, Treg). Flow cytometric fluorescent anti-human monoclonal cell surface antibodies were purchased from Beckman Coulter (Beckman Coulter, Brea, CA, USA). The details of the fluorochrome-conjugated antibodies and the schemes of the fluorochrome channels are presented in Table 2. Briefly, 100 µL of anticoagulated blood was stained with fluorescent antibodies for 20 min in the dark (room temperature). The erythrocytes were removed by adding 500 µL of OptiLyse C No-Wash Lysing Solution (Beckman Coulter, Brea, CA, USA) and incubated for 15 min in the dark (room temperature). Then, the cells were rinsed twice and resuspended in staining buffer (phosphate-buffered saline containing 2% fetal bovine serum) prior to acquisition. Strategies for CD4 + T cell subsets gating are shown in Additional file 1: Fig. S1. All samples were analysed with a 13-Color CytoFlex Flow Cytometer (Beckman Coulter, Brea, CA, USA) after daily calibration with CytoFLEX Daily QC Fluorescence (Beckman Coulter, Brea, CA, USA).

**Table 2:** Overview of the two staining panels each dedicated to a specific cell type which is indicated by individual colours

Excitation (nm)	Blue: 488				Red: 633		Violent: 405	
Emission (nm)	585	780	660	525	712	780	450	525
Fluorochrome	PE	PE-Cy7	APC	AF488	A700	A750	PB	KRO
Panel/antibodies	CD25	CD196	CD127	CD183	CD8	CD3	CD4	CD45
Volume of antibodies	20 μL	5 μL	5 μL	5 μL	5 μL	5 μL	5 μL	10 μL

*PE* phycoerythrin, *PE-Cy* phycoerythrin–Cyanine, *APC* allophycocyanin, *AF488* Alexa Flour 488, *A700* allophycocyanin 700, *A750* allophycocyanin 750, *PB* Pacific Blue, *KRO* Krome Orange

### Plasma cytokine assay

Fasting blood samples were collected in the morning. The samples were stored at – 80 °C until analysis with standard hospital assays in the central laboratory. Whole blood leukocyte counts were measured using a Sysmex XE-2100 automated haematology analyser (Sysmex Medical Electronics Co., Ltd, Japan). C-reactive protein (CRP) levels were determined using a high-sensitivity immunoturbidimetric assay (Beckman Coulter, Inc, USA). IL-6 levels were measured by electrochemiluminescence assay (Roche Diagnostics Co., Ltd, Switzerland). The concentrations of other cytokines (IL-8, tumour necrosis factor [TNF-α], interferon [IFN-γ], IL-4, IL-5, IL-17A, IL-17F, IL-21, IL-22, IL-23, IL-10, and transforming growth factor [TGFβ-1]) in serum were quantified using a Luminex-based Milliplex xMAP Human Cytokine/Chemokine Magnetic Bead Panel-Immunology Multiplex Assay Kit (Millipore, Boston, MA).

### Statistical analysis

Categorical variables are presented as numbers (percentages), normally distributed data are presented as mean ± standard deviation (SD), and nonnormally distributed data are reported as median (interquartile range, IQR) unless otherwise indicated. Continuous parametric data were analysed using Student’s t-test or one-way analysis of variance (ANOVA), while continuous nonparametric data were analysed using the Mann–Whitney U or Kruskal–Wallis test. All categorical data were analysed using a chi-square or Fisher’s exact test. All statistical analyses were performed using SPSS 24.0 (IBM, Chicago, IL, USA), and a P value < 0.05 was considered statistically significant.

## Results

### Baseline characteristics of study participants

Subjects were enrolled between May 2018 and January 2019. A total of 15 HCs, 19 HSs, 42 patients with SCOPD, and 55 patients with AECOPD were recruited. Demographic and clinical characteristics of the subjects are summarized in Tables 3 and 4. Compared to stable COPD patients, AECOPD patients had worse symptom control and pulmonary function, and higher white blood cell counts.

**Table 3:** Demographic and clinical characteristics of subjects at baseline

	HC	HS	SCOPD	AECOPD	*P* value
Patients(n)	15	19	42	55	
Age, years	62.53 ± 7.29	64.05 ± 10.99	63.55 ± 8.29	65.27 ± 06.28	0.577
Male, n (%)	13 (80)	17 94.7)	40 (95.2)	53 (96.4)	0.249
Current/Ever smoker, n	–	13/6	25/17	22 (33)	0.063
Smoking history (pack-years)^¥^	–	35 (15)	40 (22)	40 (30)	0.071
BMI (kg/m^2^)	24.21 ± 2.70	23.27 ± 2.92	22.34 ± 3.20	22.09 ± 3.94^*^	0.148
FEV_1_(L)	2.26 ± 0.47	2.50 ± 0.45	1.70 ± 1.48^*△^	0.80 ± 0.28^*△#^	0.002
FEV_1_% pred	101.13 ± 11.54	100.18 ± 16.60	54.29 ± 22.93^*△^	31.54 ± 14.12^*△#^	< 0.001
FVC(L)	2.97 ± 0.64	3.15 ± 0.63	2.96 ± 0.89	1.78 ± 0.51^*△#^	0.001
FEV_1_/FVC (%)	76.34 ± 5.11	79.67 ± 6.08	48.42 ± 12.53^*△^	45.07 ± 10.44^*△^	< 0.001
IC (L)	2.03 ± 0.45	2.11 ± 0.52	2.19 ± 0.67	1.22 ± 0.39^*△#^	0.004
IC% pred	107.94 ± 17.13	102.23 ± 28.60	86.56 ± 27.23^*△^	44.23 ± 27.00^*△#^	< 0.001
Hb (g/L)	137.67 ± 15.12	134.63 ± 11.94	151.60 ± 13.44^△^	133.65 ± 24.50^#^	< 0.001
PLT (× 10^9^/L)	194.47 ± 46.24	199.26 ± 59.40	174.55 ± 58.44	186.20 ± 66.02	0.448
WBC (× 10^9^/L)	5.42 ± 0.91	5.29 ± 1.58	5.99 ± 1.50	8.12 ± 3.58^*△#^	< 0.001
Neu (× 10^9^/L)	3.28 ± 0.88	3.26 ± 1.15	3.64 ± 1.20	6.41 ± 3.57^*△#^	< 0.001
Lym (× 10^9^/L)	1.70 ± 0.45	1.42 ± 0.61	1.68 ± 0.52	1.11 ± 0.60^*△#^	< 0.001
MONO (× 10^9^/L)	0.31 ± 0.09	0.37 ± 0.20	0.43 ± 0.15	0.50 ± 0.30^*△^	0.011
EO (× 10^9^/L)	0.11 ± 0.05	0.12 ± 0.10	0.19 ± 0.14	0.13 ± 0.20	0.169
Neu%	60.05 ± 8.79	61.33 ± 9.55	50.15 ± 7.79	74.96 ± 13.48^*△#^	< 0.001
Lym%	31.84 ± 8.29	26.74 ± 10.08	28.73 ± 7.33	16.17 ± 9.71^*△#^	< 0.001
MONO%	5.63 ± 1.36	6.78 ± 2.18	7.46 ± 1.63^*^	6.49 ± 2.30^#^	0.054
EO%	2.05 ± 1.02	2.05 ± 1.22	3.27 ± 2.37^#^	1.98 ± 3.10^#^	0.066
Neu/Lym	2.08 ± 0.83	3.66 ± 5.29	2.36 ± 1.24	9.49 ± 13.12^*△#^	< 0.001
EO/Lym	0.07 ± 0.04	0.10 ± 0.08	0.11 ± 0.08	0.10 ± 0.17	0.681

Data presented as mean ± SD unless specified. ^¥^median (IQR). *p < 0.05 vs. HC; ^△^*p* < 0.05 vs. HS; ^#^*p* < 0.05 vs. SCOPD

*BMI* body mass index, *FEV*_*1*_ forced expiratory volume in one second, *FVC* forced vital capacity, *mMRC* modified medical research council dyspnea scale, *CAT* COPD assessment test, *WBC* white blood cell, *Neu* neutrophil, *Lym* lymphocyte, *MONO* monocytes

**Table 4:** Clinical characteristics of COPD patients at baseline

	SCOPD	AECOPD	*P* value
n	42	55	
Duration of COPD, years^¥^	3 (3)	5.5 (7)	< 0.001
Cor pulmonale, n (%)	3 (7)	22 (50)	< 0.001
Home oxygen therapy, n (%)	7 (17)	42 (76)	< 0.001
Exacerbation history^¥^	1 (1)	3 (2)	< 0.001
Hospitalizations^¥^	0 (1)	2 (2)	< 0.001
Complications, n (%)			
Cardiovascular disease	4 (10)	4 (7)	0.724
Hypertension	7 (7)	7 (12)	0.772
Diabetes	1 (2)	7 (13)	0.132
mMRC dyspnoea score, n (%)			< 0.001
0	3 (7)	0 (0)	
1	13 (31)	0 (0)	
2	20 (48)	5 (9)	
3	6 (14)	19 (35)	
4	0 (0)	31 (56)	
CAT score	13.12 ± 5.74	24.60 ± 4.74	< 0.001
Using inhaled drugs n (%)			
LAMA	36 (86)	39 (60)	0.005
LABA/ICS	18 (43)	36 (66)	0.039
LAMA + LABA/ICS	15 (36)	25 (46)	0.407

Data presented as mean ± SD unless specified. ^¥^median (IQR)

*AECOPD* acute exacerbation of chronic obstructive pulmonary disease, *SCOPD* stable chronic obstructive pulmonary disease, *LABA* long-acting beta2-agonist, *LAMA* long-acting muscarinic antagonist, *ICS* inhaled corticosteroids

### Proportion of T cell subsets

**Fig. 2:**
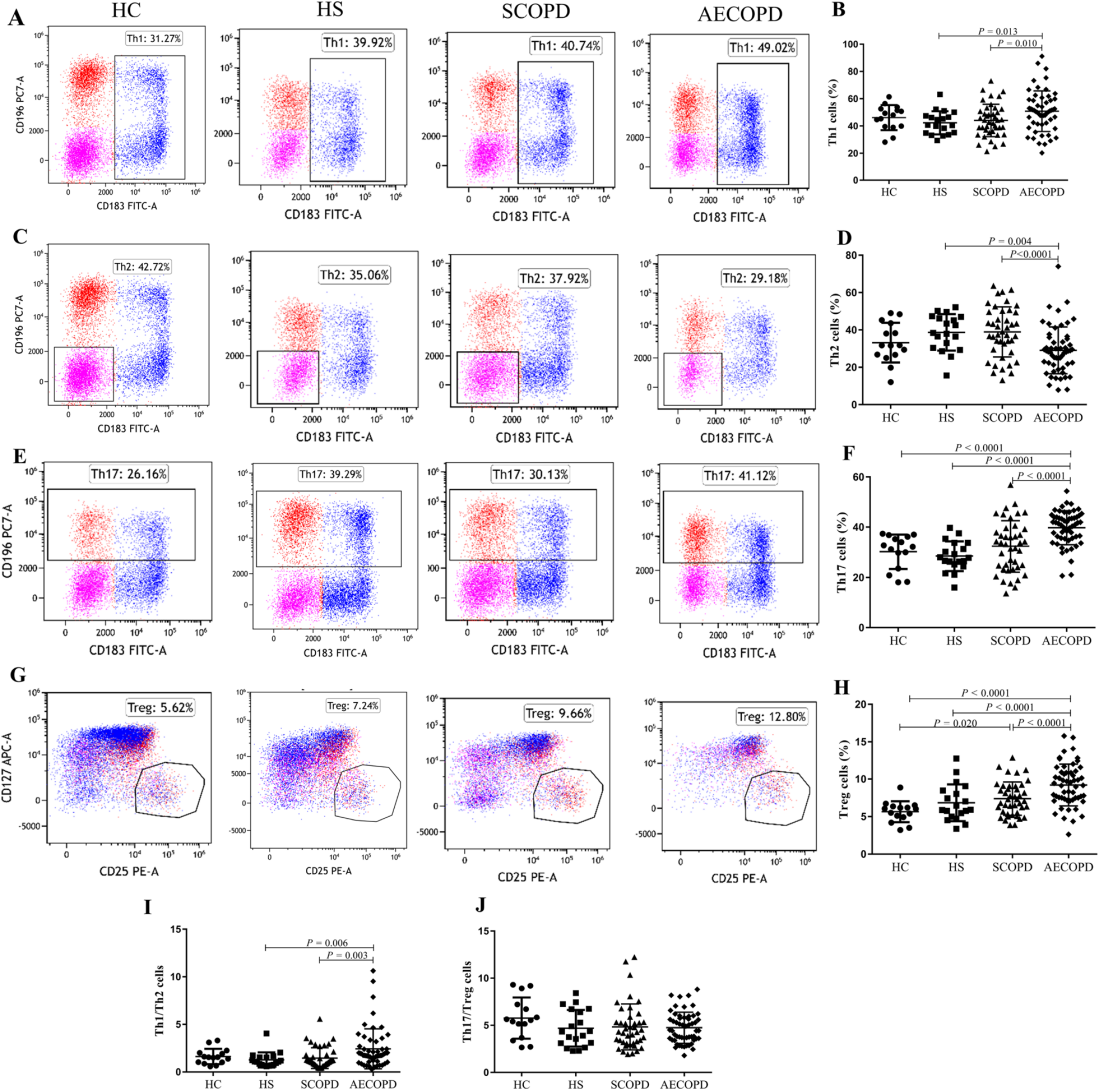
Proportion of CD4 + T cell subtypes in peripheral blood among groups. Th1 cell (**A**, **B**), Th2 cell (**C**, **D**), Th17 cell (**E**, **F**), Treg cell (**G**, **H**), Th1/Th2 cell ratio (**I**), Th17/Treg cell ratio (**J**). Data are expressed as mean number of each group (mean ± SD)

The proportion of CD4 + T cell subsets in each group are shown in Fig. 2. The proportion of Th1 cells, Th17 cells, Treg cells, and the ratio of Th1 to Th2 cells in patients with AECOPD were significantly higher than those in patients with SCOPD (p = 0.010, P < 0.0001, P < 0.0001, and p = 0.003, respectively) and HSs (p = 0.013, P < 0.0001, P < 0.0001, and p = 0.006, respectively, Fig. 2A, B, E-I). Similarly, the proportion of Th17 cells and Treg cells in patients with AECOPD was significantly higher than that in HCs(P < 0.0001 and P < 0.0001, respectively). On the contrary, the proportion of Th2 cells in patients with AECOPD was significantly lower than that in patients with SCOPD (P < 0.0001) and HSs (P = 0.004, Fig. 2C, D). There was no significant difference in the proportion of Th1, Th2, and the ratio of Th1 cells to Th2 cells among AECOPD patients, SCOPD patients, and HCs. There was no significant difference in the ratio of Th17 cells to Treg cells among four groups.

### Cytokine levels

**Fig. 3:**
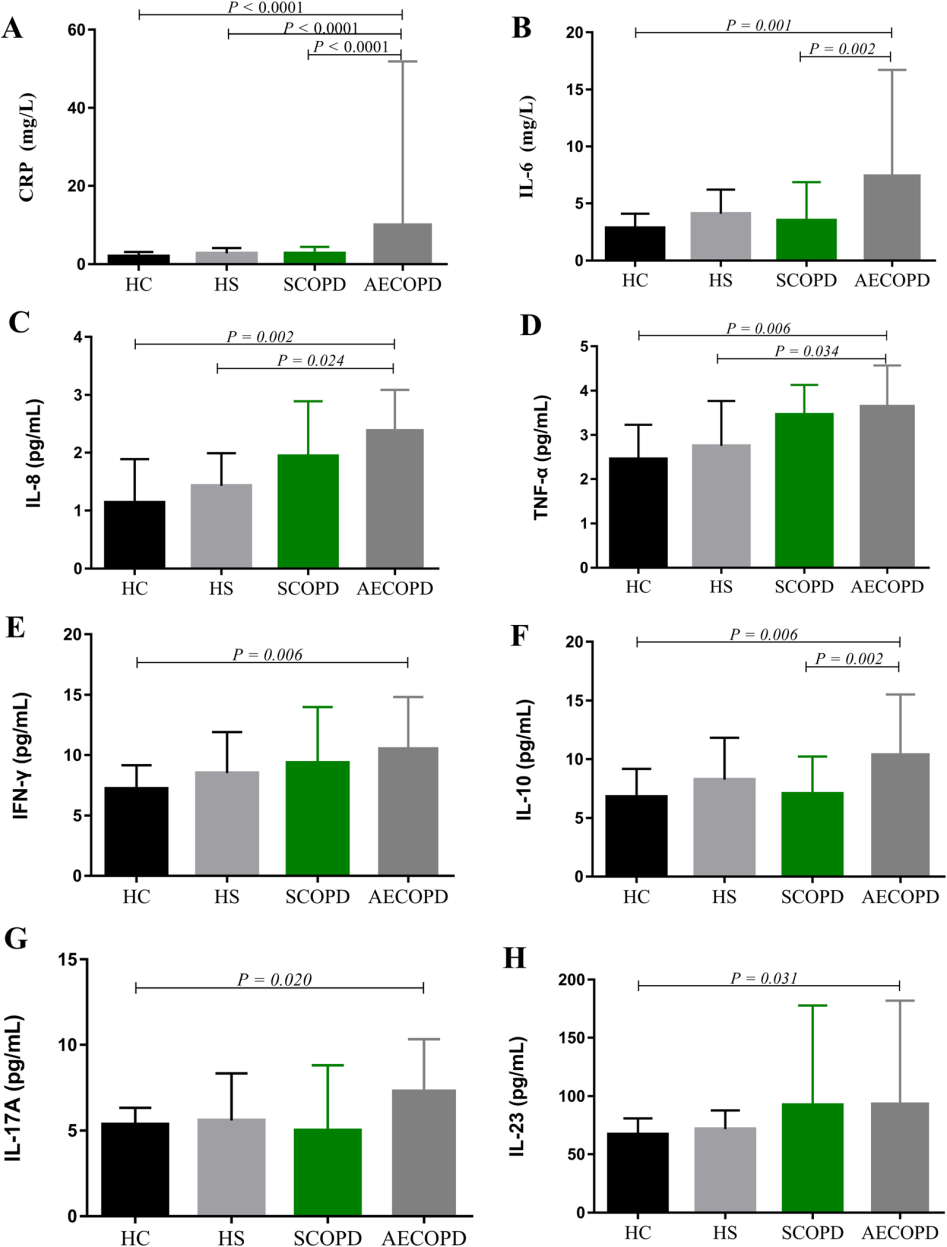
Comparison of serum cytokines in groups. CRP(**A**), IL-6 (**B**), IL-8(**C**), TNF-α (**D**), IFN-γ (**E**), IL-10 (**F**), IL-17A (**G**), IL-23 (**H**). Data are expressed as median (IQR) of each group

The levels of cytokines in all groups are shown in Fig. 3. CRP, IL-6 and IL-10 levels were significantly higher in patients with AECOPD than in patients with SCOPD (p < 0.0001, p = 0.001 and p = 0.006, respectively) and HCs (p < 0.0001, p = 0.098 and p = 0.002, respectively). Similarly, TNF-α and IL-8 levels were significantly higher in AECOPD patients than in HSs (p = 0.034 and p = 0.024, respectively) and HCs (p = 0.006 and p = 0.002, respectively). Moreover, patients with AECOPD had significantly higher levels of IL-17A, IFN-γ, and IL-23 compared to HCs (p = 0.020, p = 0.006 and p = 0.031, respectively), but not compared to patients with SCOPD and HSs. There were no significant differences in IL-4, IL-5, IL-17F, IL-21, IL-22, and TGFβ-1 levels across groups (Additional file 1: Fig. S2).

### Correlation analyses

**Fig. 4:**
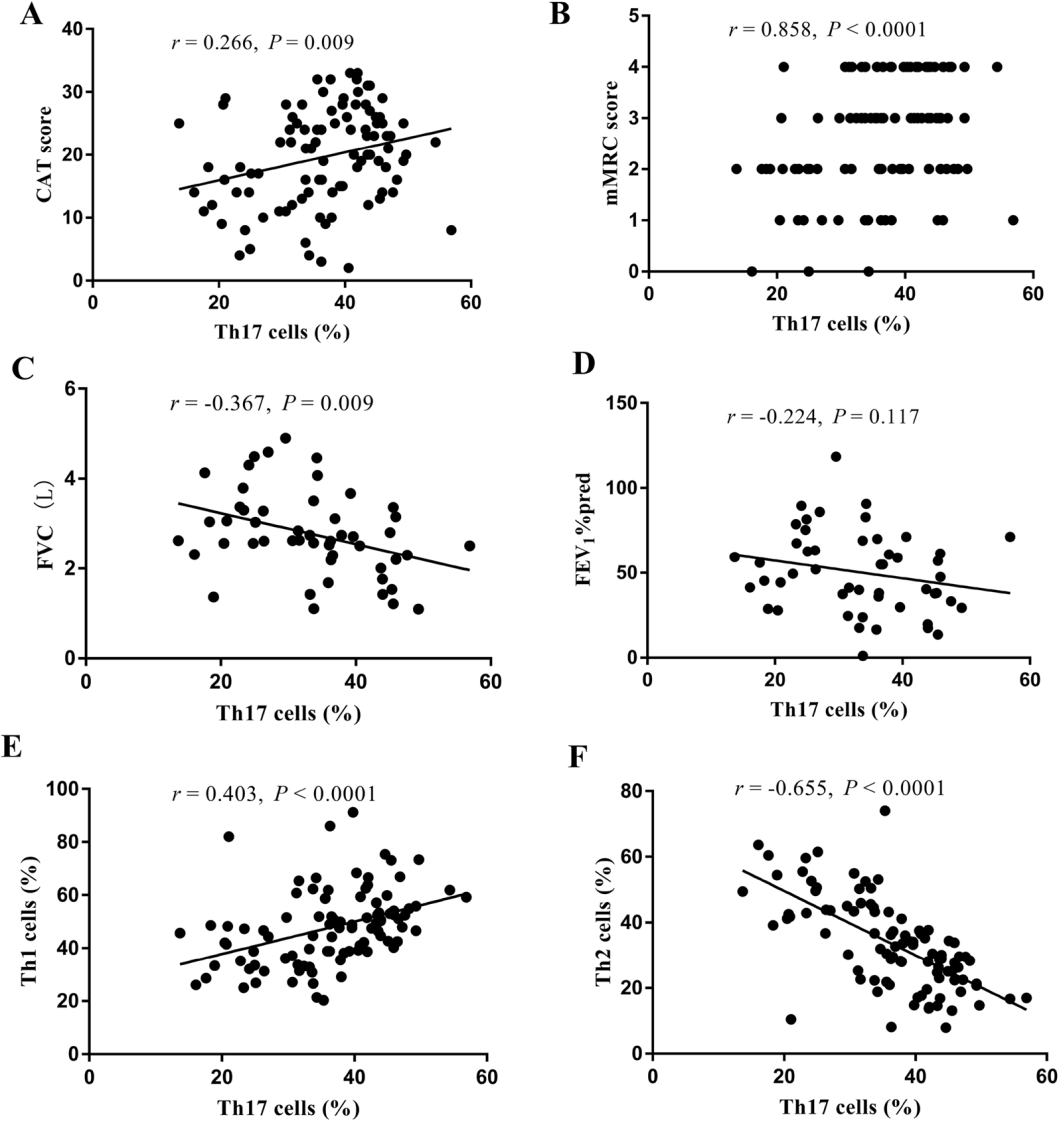
Analysis of the correlation between the proportion of Th17 cells in peripheral blood and clinical indexes. CAT score (**A**), mMRC score (**B**), FVC (**C**), FEV_1_%Pred (**D**), the proportion of Th1 cells (**E**), the proportion of Th2 cells (**F**)

Results of correlation analyses in COPD patients are shown in Fig. 4. The proportion of Th17 cells was positively correlated with CAT score (r = 0.266, P = 0.009, Fig. 4A), mMRC score (r = 0.858, P < 0.0001, Fig. 4B), forced vital capacity (FVC) (r = − 0.367, P = 0.009, Fig. 4C), and the proportion of Th1 cells (r = 0.403, P < 0.0001, Fig. 4E). On the contrary, the proportion of Th17 cells was negatively correlated with the proportion of Th2 cells (r = − 0.655, P < 0.0001, Fig. 4F). However, there was no significant correlation between the proportion of Th17 cells and FEV_1_% predicted (Fig. 4D).

## Discussion

The results of this study demonstrated that Th17 cells are positively correlated with COPD symptom score, but negatively correlated with pulmonary function index. We also characterized the immunophenotypes of patients with AECOPD, noting abnormal activation of Th1 cells, Th17 cells, and Treg cells. To the best of our best knowledge, this study is the first to describe the immunophenotype of patients with AECOPD.

There are two main methods to detect CD4 + T cell subtypes in human peripheral blood by flow cytometry. One method [19] is the traditional method of isolating peripheral blood mononuclear cells, stimulating the cells in vitro, and detecting cell surface markers and intracellular factor staining. Although this method allows for a detailed analysis of the functional characteristics of cells, it requires more blood samples, is time-consuming, and involves a large amount of cell loss. Another method[20] is to use cell surface staining exclusively to label cell surface markers such as CX-chemokine receptor 3 or CD183, CCR6 or CD196 to detect Th1, Th2, and Th17 cells; this method is a simple and rapid alternative, but these surface markers are not specific, so surface markers alone may increase the proportion of cell subsets. Acosta-Rodriguez et al. [21] found that the CD183 + CD196 + cells can produce IFN-γ and IL-17, which are also produced by Th1 and Th17 cells, respectively; therefore, we labeled Th1 cells as CD3 + CD4 + CD183 + and Th17 cells as CD3 + CD4 + CD196 + .

Th1 cells participate in the cellular immune response by secreting pro-inflammatory cytokines such as IL-2 and IFN-γ [22]. Previous studies on Th1 cells mainly focused on COPD airway inflammation, demonstrating that the expression of Th1 cell-related factors in the airway and lung tissues was increased, while there have been relatively few studies in peripheral blood. Our study found that the proportion of Th1 cells and their related cytokines in patients with AECOPD were higher than in HSs. This is consistent with the study by Sun et al. [23] which included 30 patients with AECOPD, 15 patients with SCOPD, and 15 normal controls and found higher levels of IFN-γ in AECOPD and SCOPD groups compared to the HC group, but lower IFN-γ levels in patients with AECOPD than patients with SCOPD. This inconsistency may be due to differences in patient populations between the latter study and our own; we ruled out the effects of cigarette exposure. The above results also suggest that there is a difference in serum levels of cytokines produced by Th1 cells in patients with COPD, which may be related to different stages and severity of the disease. Some scholars have shown that IFN-γ secreted by Th1 cells can induce the production and release of matrix metalloproteinase (MMP)-12 and MMP-9 in vivo; MMP can degrade the extracellular matrix components in the lung parenchyma and inhibit alpha trypsin, causing tissue cell infiltration and tissue destruction, thereby causing lung inflammation and emphysema [24]. Therefore, Th1 cells and their related cytokines contribute to the onset and development of COPD and show different characteristics in different stages of the disease; further research is thus required to elucidate the specific regulatory mechanisms involved.

Th2 cells can secrete pro-inflammatory cytokines such as IL-5, IL-6, and TNF- α, as well as anti-inflammatory cytokines such as IL-4, IL-10, and IL-13, which participate in humoral immunity and allergic responses [25, 26]. Therefore, Th2 cells are not only immune effector cells, but also have immune regulation functions. In their study of 245 patients with AECOPD, 193 patients with SCOPD and 50 HCs, Wei et al. [27] showed that the levels of IL-4 in the peripheral blood of patients with AECOPD were higher than those of patients with SCOPD and HCs. Our study found that the percentage of Th2 cells in the peripheral blood of patients with AECOPD was significantly lower than that of patients with SCOPD and HSs, while there was no significant difference in the levels of IL-4 and IL-5. A possible reason for this inconsistency may be related to the individuals selected by the specimens and the experimental methods. Therefore, the levels of Th2 cells and their related cytokines in COPD may also be related to different stages of disease development.

The ratio of Th1 to Th2 cells is relatively balanced in the normal body but can be altered in the presence of pathological changes. Previous studies have shown that the imbalance of Th1/Th2 cells is involved in the occurrence and development of COPD [28]. In both animal models of COPD and in the lung tissue of COPD patients, levels of Th1 cells and Th1-secreted IFN-γ are increased, while those of Th2 cells and Th2-secreted IL-4 are decreased. A previous study also demonstrated that patients with COPD had weaker Th2-mediated responses, including a weaker inhibitory effect on Th1 cells, and stronger Th1 cell responses, suggesting that there was an imbalance of Th1/Th2 cells in COPD patients [29]. Our study also showed that the ratio of Th1/Th2 cells in peripheral blood increased significantly in patients with AECOPD. Taken together, these results suggest that the immune mechanism of COPD involves an imbalance in the number, ratio, and function of Th1 and Th2 cells and their corresponding cytokines, and there is a Th1 cell-type immune inflammatory response in which the balance of Th1/Th2 cells is biased to Th1 cells.

Th17 cells can secrete cytokines such as IL-17A, IL-17F, IL-21, IL-22 and IL-23 which subsequently play a role in recruiting neutrophils and maintaining inflammation [13]. Previous studies have shown that Th17 cells [14, 15] and IL-17 levels [30] in the peripheral blood of patients with AECOPD are increased. These results are consistent with our own observations in patients with AECOPD and SCOPD. Th17 cells were positively correlated with COPD symptom scores (CAT and mMRC score), but negatively correlated with pulmonary function index (FVC). The latter finding is similar to that of Vargas et al. [16] who showed that the level of Th17 cells is negatively correlated with FEV_1_% predicted and FEV_1_/FVC. Therefore, there is a correlation between the level of Th17 cells and the severity of COPD; this finding has important clinical implications for COPD patients and may represent a novel index for the evaluation of COPD. In addition, our study also found that the proportion of Th17 cells was positively correlated with the proportion of Th1 cells, but negatively correlated with the proportion of Th2 cells. Some scholars found that the ratio of Th1 cells to Th17 cells increased significantly in the lung parenchyma of patients with COPD [31], while our study found that the increase of Th17 cells was mainly found in patients with AECOPD. These results suggest that there is co-activation of Th1 cells and Th17 cells in AECOPD, and these can interact to promote the onset and progression of COPD and emphysema.

Among the CD4 + T cell subtypes, there are also anti-inflammatory cell subtypes, namely Treg cells, which play an important role in immune tolerance and the maintenance of immune balance. Treg cells exert their effect mainly by producing and secreting IL-10. Previous reports on the distribution of Treg cells in COPD are not consistent. Most studies have shown that compared with HCs, the proportion of Treg cells in AECOPD [5, 6] and SCOPD [7–10] is lower. However, other studies have reported the opposite result [11, 12]. The reason for this inconsistency may be related to the number of subjects and experimental methods. There are two methods for flow detection of Treg cells: one is to stain CD4 + CD25 + FoxP3 + by breaking the cell membrane, and the other is to label CD4 + CD25highCD127-/low by cell surface staining (current study). Our study increased the sample size and took smoking into consideration, and the results showed that the proportion of Treg cells in AECOPD and patients with SCOPD was significantly higher than that of HSs and HCs. Previous researchers have proposed that the imbalance of Th17/Treg cells plays an important role in the occurrence and development of COPD [32]. Our study showed that the expression of Th17 cells and Treg cells increased in AECOPD, but the increase in Th17 cells was more significant. Therefore, the above results suggest that there is co-activation of Th17 cells and Treg cells in AECOPD, and the increase of Th17 cells is dominant. The imbalance of Th17/Treg cells plays an important role in the occurrence and development of AECOPD, but further basic research is required to elucidate the underlying mechanism.

### Limitations

This study has some limitations. First, this was a cross-sectional study and thus cannot establish causality between AECOPD and CD4 + T cell subtypes. However, it provides the basis for further prospective follow-up studies. Second, due to the inclusion of the HC group, it was ethically impossible to obtain lung tissue samples to evaluate the distribution of immune cells in the airway and lungs. Finally, patients with AECOPD had more acute exacerbations and acute hospitalizations over the past year than did patients with SCOPD. They also had more severe symptoms, as indicated by higher mMRC and CAT scores. It is speculated that the acute exacerbation and stable phase may also be related to the severity of the disease, and this needs to be confirmed in further subgroup studies with a larger sample size.

## Conclusions

Patients with AECOPD have an immunophenotype of abnormal activation of Th1, Th17, and Treg cells and an imbalance of Th1/Th2 cells favouring the Th1 cell-type immune inflammatory response. The proportion of Th17 cells was positively correlated with CAT score and mMRC score, and it was negatively correlated with FVC, indicating that there was a correlation between Th17 cells and the severity of COPD, which is expected to become an index for the evaluation of COPD severity. This study provides a basis for further studies to evaluate the mechanism of immune inflammation in AECOPD.

## Supplementary Information

**Additional file 1: Figure S1.** Gating strategy for CD4+T cell subsets. Lymphocytes were gated based on high expression of CD45 and low side scatter (SSC), and a single lymphocyte is gated by the combination of A and H signals of forward scatter (FSC), named by single cells. Lymphocytes were then classified based on CD3 expression to identify T cells, which were divided into CD4+ and CD8+ T cells. Then, according to the expression of CD196 and CD183, CD4+ T cells were divided into Th1, Th2, and Th17 cells, at the same time, CD4+ T cells were separation into Treg cells based on the expression of CD127 and CD25. **Figure S2.** Comparison of serum cytokines in groups. IL-4(A), IL-5 (B), TGFβ-1(C), IL- 17F(D), IL- 21(E), IL- 22(F). Data are expressed as median (IQR) of each group.

## Data Availability

All the data will be available to other researchers on reasonable requests to the corresponding author after publication***.***

## Abbreviations

COPD: Chronic obstructive pulmonary disease
AECOPD: Acute exacerbation of COPD
SCOPD: Stable COPD
HS: Healthy smoker
HC: Healthy control
IL: Interleukin
Th1: T helper 1 cells
Th2: T helper 2 cells
Treg: Regulatory T cells
BMI: Body mass index
CAT: COPD assessment test (CAT)
CRP: C-reactive protein
TNF-α: Tumor necrosis factor-α
IFN: Interferon
TGFβ-1: Transforming growth factor β-1
FVC: Forced vital capacity
MMP: Matrix metalloproteinase
mMRC: Modified Medical Research Council (mMRC)
