# Supplementary material for: Immunophenotype in acute exacerbation of chronic obstructive pulmonary disease: a cross-sectional study
Source: Respir Res. 2022 May 28;23:137. doi: 10.1186/s12931-022-02058-x (PMC9145461; doi:10.1186/s12931-022-02058-x)

Additional Material

Figure S1. Gating strategy for CD4+T cell subsets. Lymphocytes were gated based on high expression of CD45 and low side scatter (SSC), and a single lymphocyte is gated by the combination of A and H signals of forward scatter (FSC), named by single cells. Lymphocytes were then classified based on CD3 expression to identify T cells, which were divided into CD4+ and CD8+ T cells. Then, according to the expression of CD196 and CD183, CD4+ T cells were divided into Th1, Th2, and Th17 cells, at the same time, CD4+ T cells were separation into Treg cells based on the expression of CD127 and CD25.

Figure S2. Comparison of serum cytokines in groups. IL-4(A), IL-5 (B), TGFβ-1(C), IL- 17F(D), IL- 21(E), IL- 22(F). Data are expressed as median (IQR) of each group.


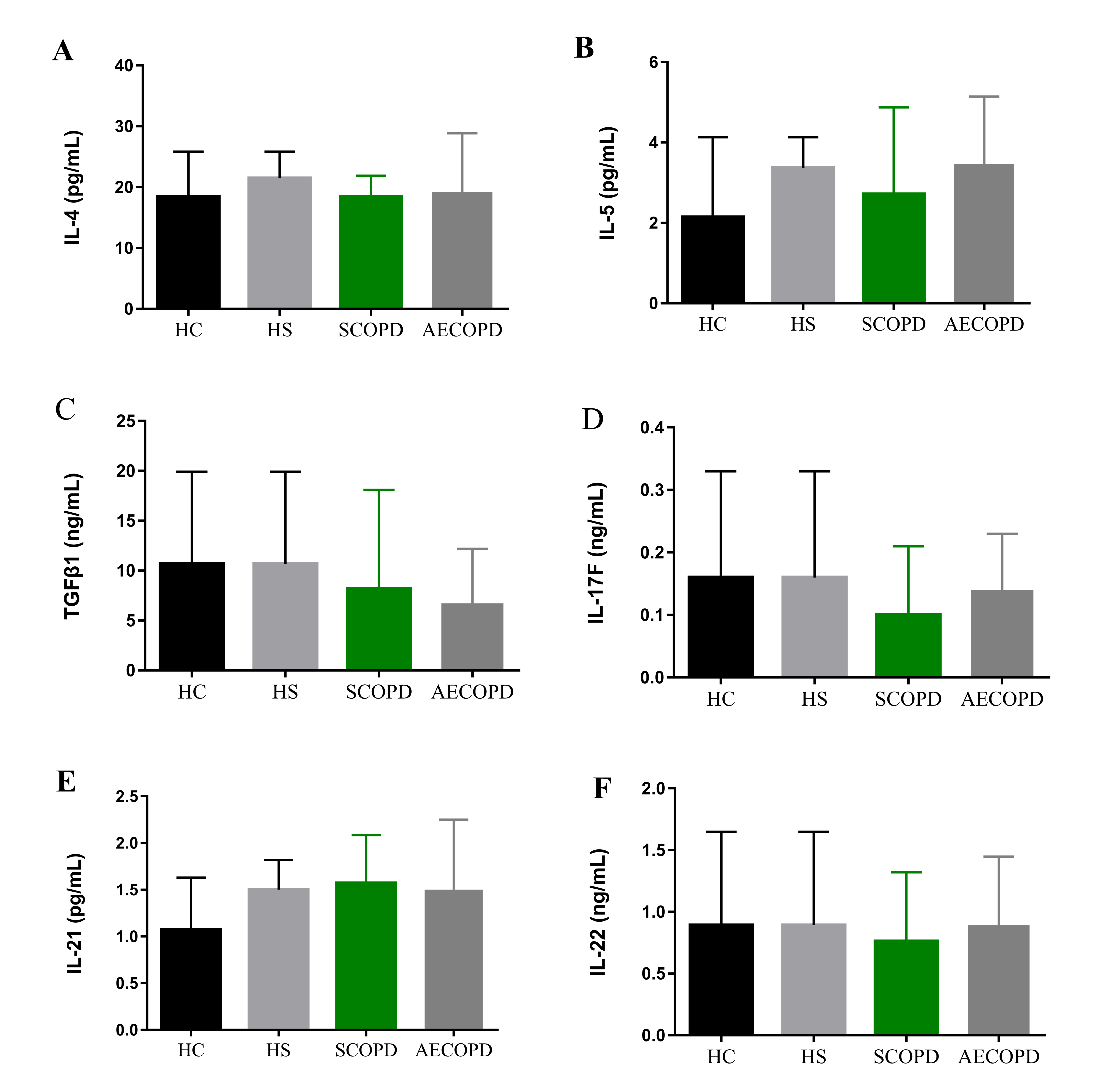

Supplement: Supplementary file 1 — Additional file 1: Figure S1. Gating strategy for CD4+T cell subsets. Lymphocytes were gated based on high expression of CD45 and low side scatter (SSC), and a single lymphocyte is gated by the combination of A and H signals of forward scatter (FSC), named by single cells. Lymphocytes were then classified based on CD3 expression to identify T cells, which were divided into CD4+ and CD8+ T cells. Then, according to the expression of CD196 and CD183, CD4+ T cells were divided into Th1, Th2, and Th17 cells, at the same time, CD4+ T cells were separation into Treg cells based on the expression of CD127 and CD25. Figure S2. Comparison of serum cytokines in groups. IL-4(A), IL-5 (B), TGFβ-1(C), IL- 17F(D), IL- 21(E), IL- 22(F). Data are expressed as median (IQR) of each group. [file 12931_2022_2058_MOESM1_ESM.doc]
